# Supplementary material for: Molecular dynamics in the stratum corneum of plantar heels in atopic dermatitis patients
Source: Biophys J. 2025 Dec 19;125(3):745–57. doi: 10.1016/j.bpj.2025.12.014 (PMC13351346; doi:10.1016/j.bpj.2025.12.014)
Supplement: Document S1. Figures S1–S4 and Table S1 [file mmc1.pdf]

Biophysical Journal, Volume *125*

Supplemental information

Molecular dynamics in the stratum corneum of plantar heels in atopic dermatitis patients

Enamul Mojumdar, Andreas Sonesson, Quoc Dat Pham, Daniel Topgaard, and Emma Sparr

SUPPLEMENTARY INFORMATION

Table S1. ^{13}C solid-state NMR peak assignments. Peak assignments with their corresponding chemical shifts are provided for all major SC lipid and protein molecular segments.

**SC lipids and protein**	**Regions**	**^{13}C segments**	**$\delta(^{13}C)$ / ppm**
Lipids (Ceramide, Cholesterol and Free fatty acids)	Chain terminal	ωCH_3	14.6
		$(\omega\text{-}1)CH_2$	23.3
		$(\omega\text{-}2)CH_2$	32.0 – 32.7
	Chain mid	$(CH_2)_n$ TG	30.5
	Chain interface	αCH_2	34.8
	Ceramide head-group	C1	55.4
		C2	61.3
		C4	135
	Chol	3	70.1
		4	42.7
		9	51.5
		14 & 17	57
		12 & 24	40.4
Protein (keratin filaments)	Terminal	Gly Cα	43.7
		Ser Cα	56.6
		Ser Cβ	62.1
		Leu Cβ & Lys Cε	40.6
		Tyr Cε	116
		His Cδ	118
		His Cε	132

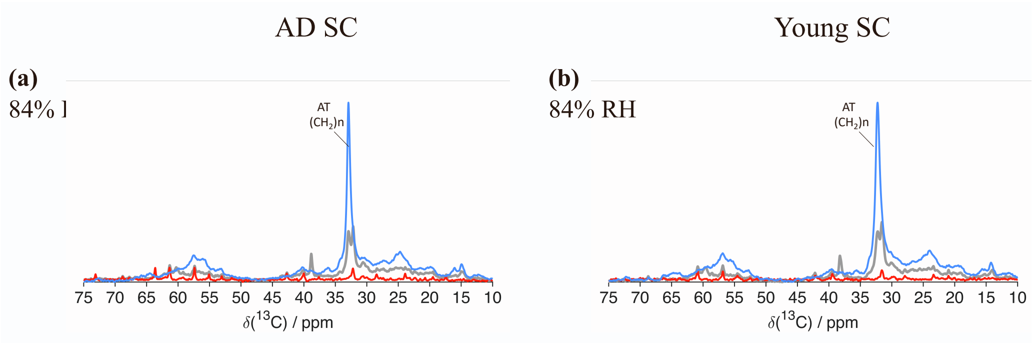

Figure S1. SC contaminated samples as observed with solid-state NMR. (a, b) SC contaminated sample from AD heel region (60-80y) and younger age group (20-30y) at 84% RH. In Figure S1a, large TG peak at approx. 30.5 ppm and other contributions in the INEPT is probably due to skin surface sebum lipids/phospholipids which are in fluid state at ambient condition. In Figure S1b, high AT peak centered around 32.5 ppm might be due to the presence of petrolatum/waxes in creams/ formulations applied onto the skin.

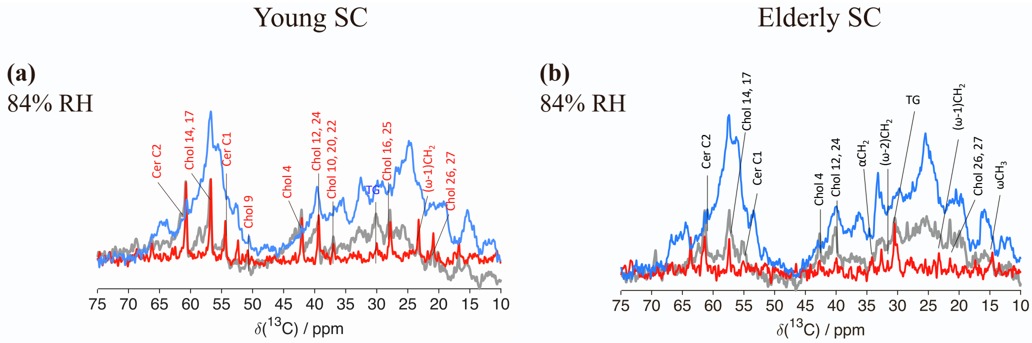

Figure S2. ^{13}C solid-state NMR study on young and elderly SC. (a, b) Samples from young and elderly SC at intermediate hydration regime corresponds to 84% RH. The individual DP (gray), CP (blue) and INEPT (red) spectrum is overlaid in all experiments for comparison purpose. The resonance lines originating from various lipid and protein molecular segments are labelled in the spectra according to the assignments provided in Figure 1 and table S1. The labelling in black in the elderly spectra (b) was used as a reference when comparing with young spectra (a). In the young spectra, the blue assignments indicate reduced signal ratio when comparing with reference for INEPT/DP signal intensities. The red labelling on the other hand indicate that a signal is increased when comparing with elderly spectra in the ratio of INEPT/DP.

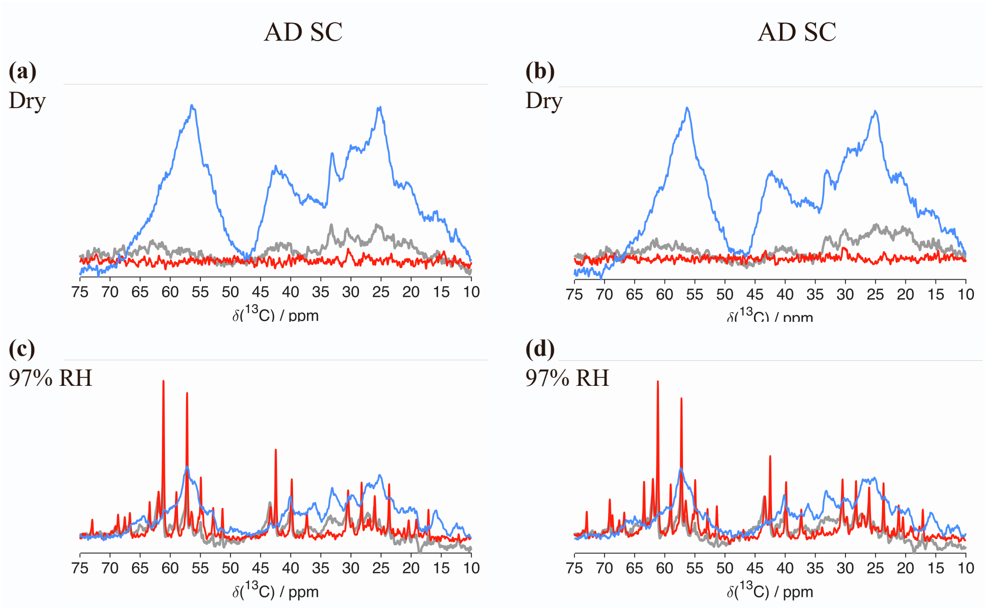

Figure S3. ^{13}C solid-state NMR study on AD SC (age 77 year). (a, c) AD sample in dry and high hydration regime corresponds to 97% RH. The individual DP (gray), CP (blue) and INEPT (red) spectrum is overlaid in all experiments for comparison purpose. The resonance lines originating from various lipid and protein molecular segments in dry and at 97% RH are very similar to the spectra shown in Figure 2 (a – dry & e – 97% RH) for AD SC and is also provided here for comparison purpose without assignments (b – dry & d – 97% RH).

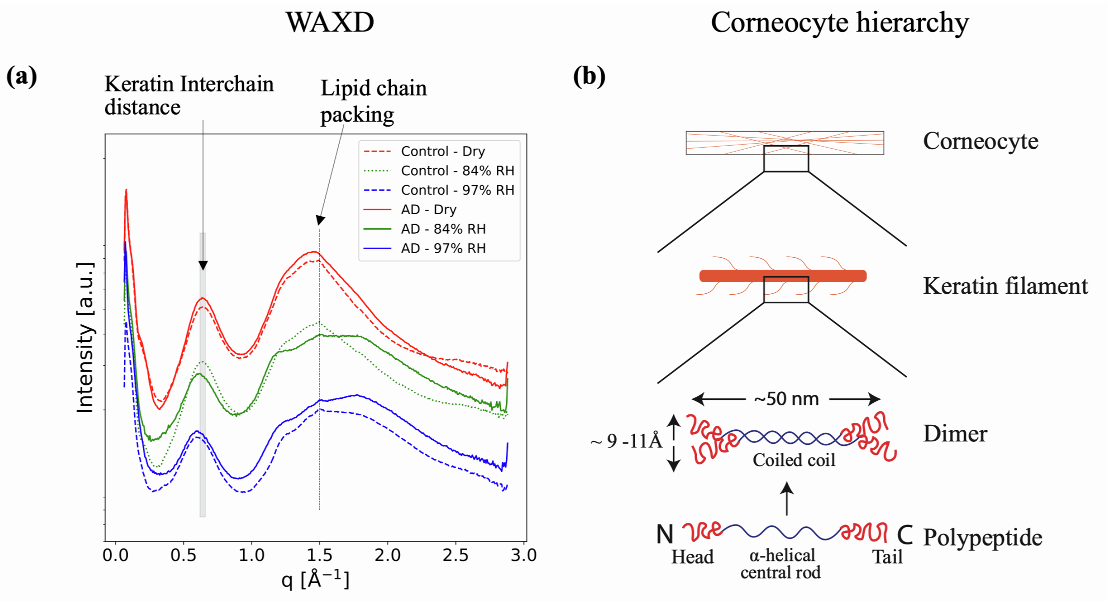

Figure S4. WAXD spectra on skin samples. (a) WAXD spectra of reference (dashed line) and AD (solid line) SC samples measured at 32 °C and at different ambient RH levels. The different colors in the spectra indicate the different hydration levels as controlled by the RH in the sample surrounding and indicated in the Figure legend. The peaks in the shaded region originate from the keratin interchain distance comprised of coiled coil dimer as depicted in (b) for corneocyte (delipidized SC) structural hierarchy. The vertical dashed line in the WAXD spectra indicate peaks arising from the lipid acyl chain packing and have not shifted due to the change in sample hydration levels.
