# Supplementary material for: Molecular dynamics in the stratum corneum of plantar heels in atopic dermatitis patients
Source: Biophys J. 2025 Dec 19;125(3):745–57. doi: 10.1016/j.bpj.2025.12.014 (PMC13351346; doi:10.1016/j.bpj.2025.12.014)
Supplement: Document S2. Article plus supporting material [file mmc2.pdf]

Biophysical Journal
Article

Molecular dynamics in the stratum corneum of plantar heels in atopic dermatitis patients

Enamul Mojumdar,[1,2] Andreas Sonesson,[3,4] Quoc Dat Pham,[1,5] Daniel Topgaard,[1] and Emma Sparr[1,*]
[1]Division of Physical Chemistry, Lund University, PO Box 124, 22100 Lund, Sweden; [2]CR Competence AB, PO Box 124, 22100 Lund, Sweden; [3]Department of Dermatology and Venereology, Skåne University Hospital, Lund, Sweden; [4]Division of Dermatology and Venereology, Department of Clinical Sciences, Lund University, Biomedical Center B14, Lund, Sweden; and [5]The Procter & Gamble Company, Reading Innovation Center, Reading, Berkshire RG2 0QE, UK

ABSTRACT The skin acts as an effective barrier against the uptake of hazardous chemicals and microorganisms as well as prevention of extensive water loss. These barrier functions are mainly assured by the outermost layer of the skin, the stratum corneum (SC). In conditions such as atopic dermatitis (AD)—a chronic inflammatory skin disease—these barrier functions can become impaired. Although AD is driven by environmental triggers and inflammation in the deeper skin layers, its effects are also evident in the SC, which is a lipid-corneocyte composite membrane. This study characterizes molecular dynamics of lipids and protein components in SC samples from the plantar heel region of AD patients aged 60–80 years, as well as healthy volunteers in the same age range and in the 20–30 age group. Using solid-state NMR, we show that, compared with age-matched healthy controls, lipids in the AD SC exhibit reduced mobility under dry conditions. With increasing hydration, mobility of both lipids and the protein keratin increases, with a stronger response observed in the AD SC. These molecular-level insights could provide further insight in the development of therapeutic strategies aimed at restoring properties of healthy skin.

SIGNIFICANCE The stratum corneum (SC) is crucial for maintaining skin barrier integrity, a function that is compromised in chronic conditions in such as atopic dermatitis (AD). Although AD pathophysiology involves deeper skin layers, the SC also exhibits significant structural and functional alterations. Here, we demonstrate that advanced solid-state NMR can detect subtle changes in the molecular dynamics of lipid and protein components in SC from AD patients and healthy individuals across different ages and hydration conditions. This approach enables the resolution of minute fluid fractions within predominantly solid SC matrix. Our findings provide detailed insight into SC molecular dynamics under diverse conditions, offering new insights into the relationship between molecular-scale alterations and macroscopic SC properties, including dryness and impaired barrier function.

INTRODUCTION

Atopic dermatitis (AD) is a chronic inflammatory skin disorder and is manifested by dry, itchy, and scaly skin conditions (1,2). Although not fully understood, the origin of AD is thought to have genetic predisposition. The AD etiology is likely due to external environmental trigger factors and inflammatory processes in the living skin tissue (1,3), whereas its effects are also evident in the dead outermost layer of the skin, the stratum corneum (SC). Among other factors involved, the multifactorial AD has been linked with filaggrin gene mutation that leads to aberrant keratinization and subsequent reduction of the natural moisturizing factor (NMF, mixture of small molecules in SC), which in turn can lead to severely dry skin (1,3–7). Factors associated with AD pathogenesis, e.g., immune dysregulation and genetic and environmental factors, also play a key role in the function and maintenance of the skin barrier functions (1,3–5,8–10). The latter observations imply that the development of the disease in lower layers of the skin has consequences in the molecular organization and properties of the SC, which is the part of skin that assures its barrier functionality (9,11).

The SC is approximately 15–20 μm in thickness and made up of anucleated keratin-filled cells, known as corneocytes, that are embedded in an multilamellar lipid matrix (12,13). The SC lipid composition is widely different from most other biological membranes in that it contains almost

Submitted August 21, 2025, and accepted for publication December 11, 2025.

*Correspondence: emma.sparr@fkem1.lu.se

Editor Name: Daniel Huster.
https://doi.org/10.1016/j.bpj.2025.12.014

© 2025 The Authors. Published by Elsevier Inc. on behalf of Biophysical Society.
This is an open access article under the CC BY license (http://creativecommons.org/licenses/by/4.0/).

no phospholipids, and the main lipids classes are ceramides, cholesterol (Chol), and free fatty acids together with trace amounts of cholesterol sulfate and other lipids (14,15). In the healthy human SC, the lipids have been found to arrange in two lamellar structures with repeat distances of approximately 6 and 13 nm, commonly referred to as short and long periodicity phase (16–18). These solid lipid lamellar phases co-exist with a small pool of disordered fluid lipids and are considered crucial for barrier function (19,20). The precise structure of the SC lipids depends on the proportion between its lipid components (18–24). It has been shown that the unique ester-linked omega-hydroxy sphingosine (EOS) ceramides with very long acyl chain are crucial to the formation of the long periodicity lamellar phase (18). In several skin diseases including AD, the amount of ceramide EOS is drastically reduced compared with healthy SC (25–27). Changes in the concentration of ceramide EOS can also influence the structure and mobility of the SC lipids (28,29). Finally, AD has also been associated with prematuration and/or aberrant differentiation of corneocytes (9,30), which may in turn imply molecular alterations in protein assembly within the corneocytes.

The macroscopic properties of the SC, such as barrier function, mechanical properties, and its ability to hold water, may all be altered by changes of the molecular structure and fluidity of the SC lipid and protein components. Such changes can be induced by changes in the molecular composition as implicated for several skin diseases (28,31–33), by changes the skin surroundings (34–36), and by the uptake of chemicals in the skin (36,37). Hydration plays an important role in fluidizing a fraction of SC lipids (35,38), which can lead to increased skin permeability of drugs and other chemicals (39,40). Changes in skin hydration will also affect the properties of the keratin filaments inside the corneocytes (34,35), which is considered important in regulating skin elasticity and plasticity strain (41–44). To our knowledge, it is not established whether the corresponding hydration response in SC lipids and proteins also takes place in the AD SC. Finally, the mixtures of small polar molecules from NMF and skin care products can act to maintain the balance between fluid and rigid SC lipid and protein components in conditions of mild or severe drying (45,46). It is therefore predicted that AD-related reduction in NMF will impact the fluidity of lipid and protein components with possible consequences on skin macroscopic properties.

In the present study, we characterize structural and dynamical differences in the lipids and protein molecular components in SC samples taken from the planter heel region of AD patients aged 60–80 years, as well as healthy volunteers in the same age range and those aged 20–30 years. We exploit a highly sensitive solid-state NMR method to characterize differences in the molecular mobility of the SC lipid and protein in the different SC samples (47,48). This technique has been recently successfully used in studies of porcine SC in different hydration, temperature, and pH conditions as well as SC treated with different chemicals relevant for NMF and cosmetic and pharmaceutical formulations (35,38,49–51). Based on the NMR data, we have been able to delineate tiny fluid fractions from a majority of solid SC materials that are not accessible with other commonly used methods, such as x-ray diffraction. The NMR data on SC mobile components thereby provide a valuable complement to the extensive literature focusing on characterization of solid SC components (16,29,52,53). All the SC samples were investigated at different hydration conditions by being exposed to either dry air or air with a controlled relative humidity (RH). The results provide insights into the SC molecular dynamics at various skin conditions and may contribute to the understanding of how molecular changes impact macroscopic properties of diseased and aged skin. The understanding of the differences in SC molecular dynamics between healthy and diseased SC and its connection to the macroscopic skin properties may further support the development of treatments to limit disease-related skin dryness and impaired skin barrier function. In particular, it can stimulate approaches that focus on regaining molecular or material properties (here, fluidity) similar to healthy skin that do not rely on restoring the exact composition of the healthy system. Such approaches may include the uptake of, for example, small NMF-like molecules or fatty acids into the SC (36,46).

MATERIALS AND METHODS

Materials

Chloroform, methanol, and ethanol were purchased from Sigma-Aldrich Chemie (Schnelldorf, Germany). KCl and K_2SO_4 were obtained from Merck. Water was of Millipore quality produced by Milli-Q water filtration system with a resistivity of 18 MΩ cm at 25°C.

SC sample collection

All the samples were collected from the heel region of patients and healthy volunteers who had been advised not to use any creams or lotions on their skin for at least a day before skin sampling. For the sample collection procedure, the skin was first quickly wiped with an ethanol-soaked cotton swab. Care was taken not to use large volumes of ethanol or long exposure as the penetration of ethanol has been shown to affect SC lipid and protein components (36). To avoid interference from the ethanol exposure, the skin was then tape-stripped three times with Pressure Sensitive Tape (Scotch, 3M, Sweden) to remove the very outer SC layer. The SC samples were then collected by use of curettage, performed with disposable 7-mm ring dermal curettes (kai Europe, Solingen, Germany). In control experiments performed on SC samples collected without prior washing and tape stripping, traces of contamination, e.g., squalene, unsaturated lipids, and other sebum lipids could be detected by means of NMR (Fig. S1).

The study was approved by the regional ethics examination board of Lund, Sweden (entry no. 344/2016), and the participants gave informed consent complying with the Helsinki Declaration.

Sample preparation

All the samples were first dried in a vacuum desiccator in the presence of water absorbing silica gel and subsequently stored in the freezer at −20°C until

further use. The samples consisted of powder and small flakes. Previously, we showed that there is no detectable difference of NMR spectra obtained for pulverized SC (small flakes) and intact sheets of SC, as long as the sample treatment is the same (54). It is however noted that the time needed to reach stable (close to equilibrium) state with respect to hydration is shorter for the pulverized sample compared with the SC sheets. The SC samples were taken out from the freezer and kept at room temperature for about 30 min before any further treatment in order to avoid condensation of water on the SC powder.

To prepare samples for NMR measurements, approximately 20 mg of dry samples was used. The samples were equilibrated at the given RH for ca. 24 h in a closed desiccator placed in an oven at 32°C. For each sample, three different hydration conditions were chosen: dry (close to 0% RH), intermediate hydration (84% RH), and close to fully hydrated (97% RH) conditions. The hydration levels were chosen based on previous studies where we expect to see differences in the lipid and protein signals in the NMR spectra, where the intermediate conditions (84% RH) are close to the threshold hydration condition for protein mobility (34,55). The samples were equilibrated in a desiccator at controlled RH, set by the vapor pressure above aqueous solutions saturated with either KCl (84%) or K_2SO_4 (97% RH). For the preparation of the dry SC, the desired amount of vacuum-dried material was put in a paraffin-sealed Eppendorf tube and kept in the oven at 32°C for 24 h for temperature equilibration. After equilibration, the samples were quickly transferred (within a minute) to an NMR insert (Bruker) with sealed screw to avoid hydration/dehydration. Later, the screw-tight insert was placed in a 4-mm MAS NMR rotor (Bruker) with cap on it and placed in the NMR magnet for measurements. The samples used for the wide-angle x-ray diffraction (WAXD) experiments (5–7 mg each) were prepared in the same way as for NMR. After equilibration, the samples were quickly transferred (within a minute) to screw-tight sandwich cells with mica sheets in between.

Study design

As each NMR experiment takes approximately 9 h and requires ca. 20 mg of SC sample, it is not feasible to do screening studies with several replicates to obtain good statistics. In order to reduce the effects of biological variations in the comparisons between SC from different age groups, we merged SC samples from different individuals belonging the same age group into one "batch." The SC batches for healthy SC from younger individuals (20–30 years old) and older individuals (60–80 years old) contain sample from four different individuals, including both males and females. In order to check the variability, we compared the results from these batches with SC from one additional individual from the same age group, showing good reproducibility (Fig. S3). For the samples from patients with AD, we repeatedly noticed contamination in the SC batches, which was most likely due to prior treatment with skin care products (Fig. S1). This then led to contamination of the whole batches, which were then discarded. For the studies of AD SC, we therefore discarded the samples made from the contaminated batches and instead studied samples from different individuals separately, where SC from each individual was exposed to different RHs. This was then repeated for samples from different individuals to check for reproducibility (Fig. S3). A summary of all samples that were judged to be free from severe contaminations is listed in Table 1.

Preparation of delipidized SC

Delipidized SC was prepared from the AD SC samples listed in Table 1 using the method adopted from (56). In brief, approximately 30 mg of the powder samples was placed in 25-mL glass vials. Delipidization of SC was performed using chloroform:methanol solutions in three different mixtures, namely 2:1, 1:1, and 1:2 v/v ratios (45). Each extraction step was carried out at room temperature and performed for about 2 h under gentle shaking. The nondissolved material was collected after each step by filtration. After all three steps of solvent extractions, the filtered material was soaked in methanol overnight. Finally, the methanol filtered delipidized SC was thoroughly rinsed with Milli-Q water several times in order to get rid of all the methanol and subsequently dried under vacuum in a desiccator in the presence of water absorbing silica gel. It is here pointed out that the current extraction method removes extracellular lipids but not the lipid layer that is covalently bound to the corneocytes (57).

Solid-state NMR

NMR experiments were carried out on a Bruker Avance AVII 500 NMR spectrometer. The instrument was equipped with Bruker E-free 4-mm magic angle spinning (MAS) probe, operated at 5 kHz frequency and with the ^{1}H and ^{13}C resonance frequencies of 500 and 125 MHz, respectively. The temperature was set to be 32°C and calibrated by using methanol. The present solid-state NMR is a combination of three individual experiments performed on the same sample in a sequential manner. The three experiments are denoted as direct polarization (DP), cross polarization (CP) (58), and insensitive nuclei enhanced by polarization transfer (INEPT) (59). All the ^{13}C NMR spectra were obtained under 68-kHz two-pulse phase modulation (60) ^{1}H decoupling. ^{1}H and ^{13}C hard pulses were given at $\omega_1^{H/C}/2\pi = 80$ kHz. Ramped CP (61) was performed with ^{13}C nutation frequency set to 80 kHz and the ^{1}H nutation frequency linearly ramped from 72 to 88 kHz during 1-ms contact time. For refocused INEPT (62), the delays $\tau = 1.8$ ms and $\tau' = 1.2$ ms were used. By using these acquisition parameters, INEPT yields signal for C–H bonds with reorientational correlation time $\tau_c < 10$ ns and orientational order parameter $|S_{CH}| < 0.2$ (48,49). CP is essentially complementary to INEPT for which signal is observed when $\tau_c > 0.1$ ms. However, in the case of anisotropic liquid with $\tau_c < 10$ ns and $|S_{CH}| \approx 0.1$, both CP and INEPT are efficient, and in the intermediate regime, $\tau_c \approx 1$ μs, only CP signal is observed. In the present work, depending on the presence of CP and INEPT signals, the definitions "rigid" and "mobile" molecular segments have been introduced. These definitions can be interpreted in terms of C–H bond reorientational parameters, τ_c and $|S_{CH}|$, as stated above. A spectral width of 250 ppm was used, and the number of scans per experiment was 2048, with acquisition time and recycle delay being 0.05 and 5 s, respectively. This gives a total estimated time of approximately 9 h for the three experiments. The methylene signal of solid α-glycine at 43.67 ppm was used to calibrate the ^{13}C chemical shift scale. Data processing was done using a line broadening of 20 Hz, zero filling from 1597 to 8192 time domain points, Fourier transformation, phase correction, and baseline correction by using in-house MATLAB code partially derived from matNMR (63).

Wide angle x-ray diffraction

WAXD studies were performed using an in-house x-ray setup, SAXSLab Ganesha 300XL instrument (SAXSLAB ApS, Skovlunde, Denmark), equipped with 2D 300K Pilatus detector (Dectris, Baden, Switzerland). The scattering intensity (I) was recorded as a function of scattering vector q in reciprocal angstrom (Å). q is defined as $q = \frac{4\pi \sin\theta}{\lambda}$, where θ is the scattering angle, and λ is the wavelength of the incident x-ray, which is 1.54 Å in the present experiment. The two-dimensional (2D) scattering pattern recorded by the detector was radially averaged using the software SAXSGui to obtain 1D I versus q data. The d-spacing was then calculated from $d = \frac{2\pi}{q}$. The scattering data were recorded for about 20 min, and the temperature was controlled using an external circulating water bath to 32°C.

RESULTS

In the present study, we investigate differences in lipid and protein molecular dynamics in SC harvested from the

TABLE 1 Summary of SC samples investigated

Notation	Abbreviation/Meaning	Skin Type	Age Group	No. of Volunteers	Gender
AD	Atopic dermatitis	Lesion	60-80 years	2	1 male and 1 female
Reference/elderly	Reference – aged skin	Healthy	60-80 years	4 (batch) +1	2 males and 2 females
Young	Reference – young skin	Healthy	20-30 years	4 (batch) +1	2 males and 2 females

All samples were collected from the plantar heel area of skin from White individuals. The samples were classified with respect to their age group (60–80 years old or 20–30 years old) and with respect to the skin disease atopic dermatitis (AD). The AD SC belongs to the age group 60–80 years old, and the samples from heathy volunteers in this age group are therefore denoted as a reference sample in the evaluation of data of the SC from AD patients.

plantar heel region of AD patients and from healthy individuals of different age groups. Details of SC samples with number of volunteers, their age, and gender are given in Table 1. For all types of SC samples, we study the effects of changing the hydration conditions, spanning from dry to close to full hydration conditions. Below, we first describe the NMR methodology. We then present experimental data for AD SC. In all comparisons between SC from AD patients and healthy individuals, we use samples collected from individuals within the same age group (60–80 years). Finally, we study the differences in SC molecular dynamics for SC from healthy individuals in different age groups. All the SC samples were collected at the hospital. Care was taken to avoid contamination from sebum lipids, skin care products, or other chemicals from surroundings. Samples in which we detected traces of contaminations from, for example, skin care products were discarded, and examples of two such contaminated spectra are presented in Fig. S1 for AD and control SC samples. A replicate for noncontaminated AD SC sample is presented in Fig. S3.

Solid-state NMR to unravel molecular dynamics of intact SC

We use solid-state NMR to investigate the molecular mobility in different molecular segments of the SC lipids and protein components. The NMR method is highly sensitive to small changes in the minor fluid fraction of SC components, and it simultaneously detects solid/rigid components in the same sample. In previous studies, we managed to assign the chemical shifts of all major peaks in the crowded ^{13}C spectra to specific carbons in lipids and amino acids in intact porcine SC (35). The major peaks assigned for the human SC samples are labeled in Fig. 1 *b* and *c* and presented in Table S1. Thanks to the high sensitivity to variations in molecular dynamics with close to atomic resolution, the method is a powerful tool to study fluidity in intact SC and thus complement the extensive literature that is mainly focused on characterization of solid SC components (16,18,53,64). The present solid-state NMR method consists of DP, CP, and INEPT (58,59). CP and INEPT are common schemes for ^{1}H to ^{13}C polarization transfer for solid and mobile samples, respectively (Fig. 1 *b–d*). The DP (Fig. 1 *d*, gray) experiment captures all naturally occurring ^{13}C signals from both solid and mobile components present in the sample without the need for polarization transfer and could serve as a reference for peak assignment (35). This implies that the mobile components would be more noticeable in the DP signal due to their smaller line widths. For comparison purposes, the DP, CP, and INEPT spectra are then overlaid (Fig. 1 *d*). Mobility is herein defined with respect to the reorientational dynamics of the C−H bonds of the different molecules. The INEPT spectrum (Fig. 2 *c*, red) shows a signal for "mobile" segments (defined as segments with rotational correlation time $\tau c < 10$ ns), whereas the CP experiment (Fig. 1 *b*, blue) boosts the signal for "rigid" segments with longer correlation times or with anisotropic reorientations (49). Under the present experimental conditions, the rigid components in the sample are attenuated by incomplete ^{13}C T1 relaxation even at d1 = 5 s. The broad CP spectrum in the cartoon in Fig. 1 *b* shows that the segment studied in this imaginary sample is predominantly rigid, whereas the sharp INEPT in Fig. 1 *c* indicates that a fraction of this segment is present in mobile state. A detailed description of the method is given elsewhere (49).

To facilitate the reading and interpretations of the NMR spectra from the SC samples, we translate the data in the spectra to simplified cartoons that indicate the mobility in SC lipids and keratin proteins (Fig. 1 *b–d*). The drawings in blue represent rigid lipids and protein segments, whereas red indicates mobile segments in these molecules. Depending on the chemical shift, we can assign which region of the lipids and which amino acids that are mobile. All NMR data are also summarized in Table 2, where we distinguish the effects on different parts of the ceramide and fatty acid chains: the terminal (ωCH_3, (ω-1)CH_2, and (ω-2)CH_2 segments), the middle part $(CH_2)_n$, and the part close to the headgroup (αCH_2). Based on the chemical shift, we can also distinguish between all-trans conformation and trans-gauche conformational isomerization of the chain. Table 2 also contains information on mobility in the ceramide (Cer) headgroups (C1 and C2, Fig. 1 *e*), Chol molecular segments 4, 9, 12, and 24 (Fig. 1 *e*), and some amino acids that are abundant in the keratin filaments: glycine (Gly), serine (Ser), tyrosine (Tyr), and histidine (His) (Fig. 1 *f*).

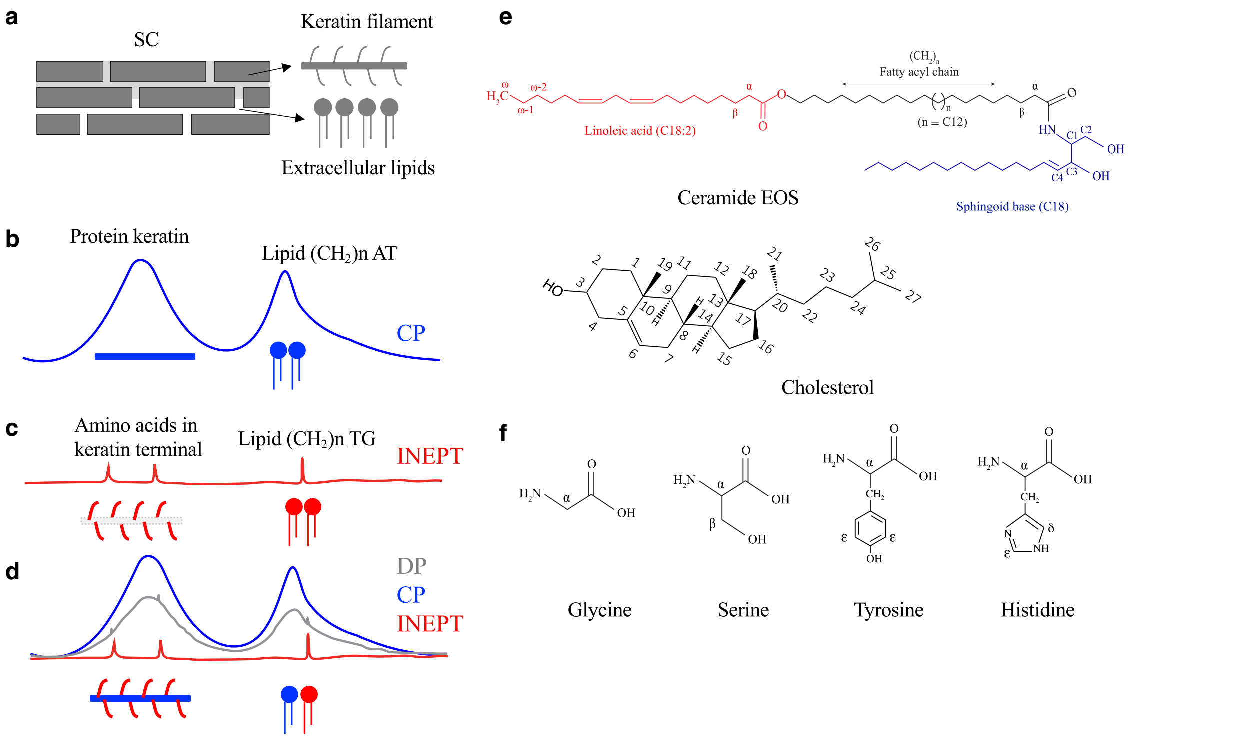

FIGURE 1 Schematic representation of SC and its constituents. (*a*) A superficial thin layer of the skin epidermis, known as stratum corneum (SC), which is approximately 15–20 μm thick. The SC consists of dead corneocyte cells that contain rigid keratin filaments. The protruding terminals of keratin filaments are enriched in amino acids serine and glycine, whereas the core contains abundantly leucine and lysine. The cells in the SC are embedded in a lipid matrix. The extracellular lipid matrix consists of mainly ceramides, cholesterol, and free fatty acids and organizes in a multilamellar stack. (*b–d*) Cartoon presentation of the solid-state NMR method used in this study. DP (*gray*, *d*) detects all carbons in a sample. CP (*blue*, *b*) provides signals only for those carbons that are present in the rigid state. INEPT (*red*, *c*) provides signals only for mobile carbons. All the spectra are overlaid and presented for comparison purpose (*gray*, *blue*, and *red*, *b–d*). Schematics of SC keratin filaments and lipids are also presented in a different color scheme for better understanding. The blue color in the protein and lipid segments indicates solid segments, whereas red indicates mobile segments. (*e*) A representative molecular structure of SC lipids, including ceramide and cholesterol with their carbon numberings in various parts of the molecules. (*f*) Molecular structure of glycine, serine, tyrosine, and histidine that are enriched in the protruding terminal of keratin filaments within the corneocyte with their carbon labels.

Molecular mobility of AD SC extracellular lipids

Fig. 2 shows solid-state NMR spectra of AD and reference SC in dry and humid conditions. The AD SC sample shows the overall feature of a rigid material, but there are some segments that are mobile for most conditions investigated, as inferred from the INEPT (red) signal. For the dry AD SC sample, mobility was only detected for the central part of the lipid hydrocarbon chains (Fig. 2 *a*). When humidity was increased to 84%, the AD SC became more fluid compared with the dry state, showing lipid mobility for both hydrocarbon chain segments close to the headgroup and the terminal parts, as well as the Cer headgroups and Chol (Fig. 2 *c*). When the RH was further raised to 97%, the mobility further increased for all lipid segments (Fig. 2 *e*). NMR spectra for additional AD samples are provided in Fig. S3 along with the spectra presented here for comparison purpose.

The results obtained for the SC lipids from AD patients can be compared with corresponding NMR data from healthy SC in the same age group (here named reference SC) (Fig. 2; Table 2). For the dry and intermediate (84% RH) hydration state, the overall the fraction of mobile chains is lower in the AD SC compared with the reference SC. This applies to both the central parts of the lipid chains as well as segments close to the lipid headgroup. At the highest RH investigated (97%, Fig. 2 *e* and *f*), the fraction of mobile chains was similar in the AD and references SC samples. From the spectra in Fig. 2 *c–f*, it is further concluded that for high RH, the INEPT signal originating from the Cer headgroups and the Chol was higher in for the AD SC compared the reference SC, inferring higher molecular mobility in these segments. Finally, in the higher chemical shift range of the NMR spectra (100–150 ppm) (Fig. 2 *g* and *h*), we observed additional signals from carbon segments in the lipids, again showing higher mobility in the Cer headgroup in the AD SC compared with the reference SC.

Molecular mobility of AD SC amino acids

The keratin-filled corneocytes constitute ca. 85% of the total weight of dry SC (52) and are considered crucial to the

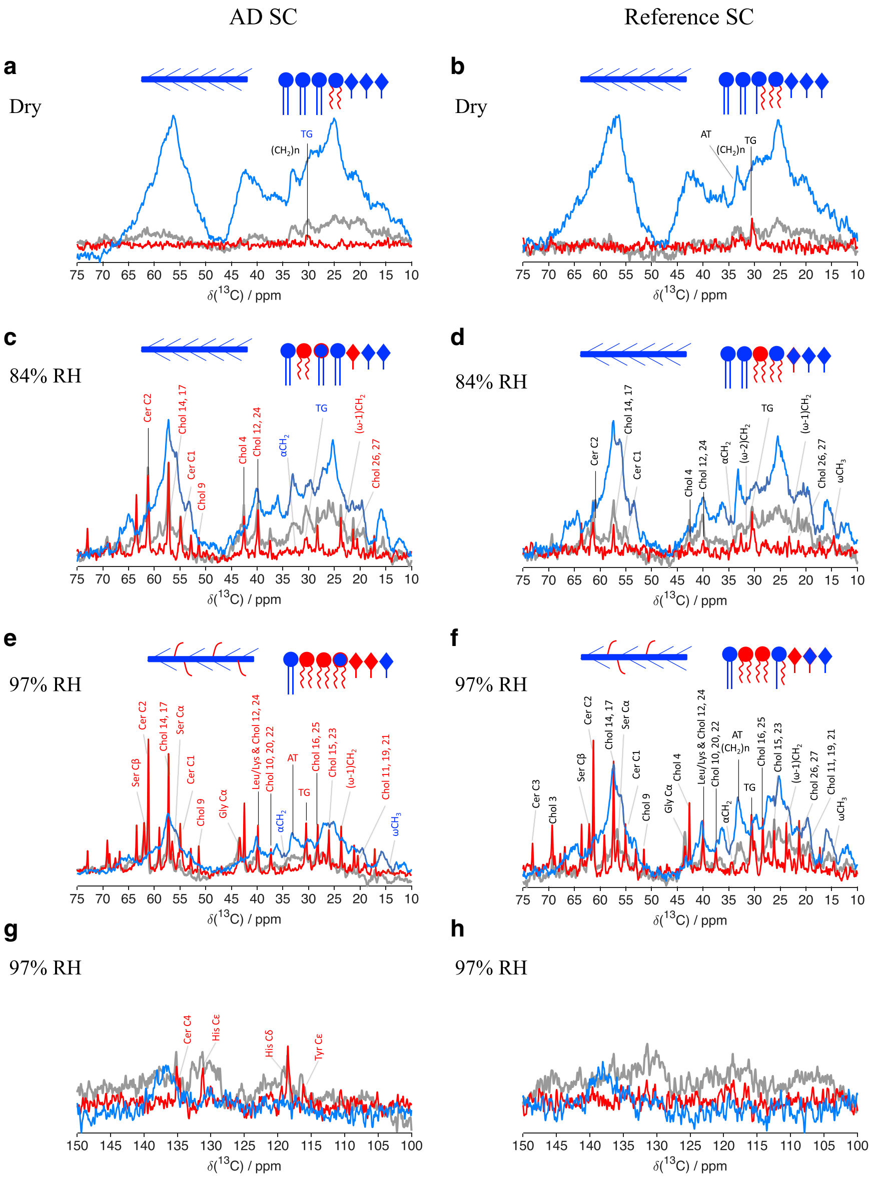

FIGURE 2 ^{13}C solid-state NMR spectra from AD and reference SC samples. The experiments were performed at three different hydration conditions: dry (close to 0% RH), intermediate (84%RH), and high (97% RH) humidity. The individual DP (*gray*), CP (*blue*), and INEPT (*red*) spectra are overlaid for comparison purpose. Peak assignment refers to the labels in Fig. 1 and Table S1. The labeling in red in the AD spectra (*a*, *c*, *e*, and *g*) indicates an increased in INEPT/DP signal ratio compared with the corresponding reference sample at the same RH (*b*, *d*, *f*, and *h*). Analogously, blue labeling indicates reduced INEPT/DP signal ratio compared with the reference sample. The red labeling in the high ppm (100–150 ppm) region of the AD spectra (*g*) at 97% RH denotes amino acid signals from the keratin proteins that weren't seen in the reference SC spectra (*h*). To facilitate interpretation, cartoon representations showing molecular mobility and rigidity to the lipids and protein molecular components are shown on top of the spectra. In the cartoons, blue and red indicate rigid and mobile molecular segments as evident from the CP (*blue*) and INEPT (*red*) spectra, respectively. The Cer and Chol headgroups in the cartoon are represented as round and diamond shapes, whereas straight and wiggly lines indicate rigid and mobile chain segments, respectively. Replicate spectra for AD SC at both dry and 97% humidity conditions are also presented in the Fig. S3 along with the spectra presented here (*a* and *e*).

mechanical properties of the SC (65–67) as well as the SC ability to take up and hold water (68). Overall, the corneocytes have a polar interior due to the presence of hydrophilic groups, e.g., –OH, –NH, –COOH, of the amino acids exposed at the keratin filaments. When the skin is present in a humid environment, the corneocytes take up substantial amounts of water (69), which may lead to changes in structure and molecular mobility of its constituents (34). In the NMR spectra in Fig. 2, we identify several peaks that can be assigned to keratin amino acids. The prevailing broad CP signal (blue) at 50–70 ppm implies that the majority of all amino acid molecular segments are rigid under all conditions investigated. At 97% RH, a small INEPT signal is detected at chemical shifts corresponding to the amino acids serine (Ser Cα and Ser Cβ at ∼57 and 62 ppm, respectively) and glycine (Gly Cα at ∼44 ppm). These amino acid residues are abundant in the protruding terminals of keratin filaments (35). Serine and glycine are also present in SC as free amino acids as part of the NMF (70).

The NMR data in Figs. 2 and 3 show overall a similar behavior in terms of amino acid molecular mobility for the AD and the reference SC. For both samples, the response to hydration is similar to previous observations for porcine SC at varying water contents (34,35). Still, there are some significant differences between the human SC samples. There is slightly higher mobility for Ser residues in the sample from AD SC compared with the reference SC sample (Fig. 2 *e* and *f*). Furthermore, inspection of the spectra at higher chemical shifts (Fig. 2 *g*) reveals signals from mobile carbons (approximately 116, 118, and 132 ppm) in the AD SC that cannot be resolved for the healthy reference SC (Fig. 2 *h*). The chemical shifts of the latter peaks are

TABLE 2 Summary of solid-state NMR data on molecular dynamics in samples from AD SC and SC from healthy volunteers in different age groups

**Lipid**						**Protein**		
	**Chain Terminal**	**Chain mid**	**Chain interface**	**Ceramide head**	**Chol**	**Gly, Ser, Tyr & His**		
Carbon segments	ω, (ω-1) & (ω-2)	TG	α	C1, C2 & C4	C4, C9, C12 & C24	Gly Cα	Ser Cα & Ser Cβ	Tyr Cε, His Cε & Cδ
δ(ppm) →	14.6, 23.3 & 32.5	30.5	34.2	55.4, 61.3 & 135	42.7,51.5, 40	43.6	56.6, 62.1	116, 132 & 118
Dry - SC samples of healthy & diseased volunteers								
AD (60-80y)								
Ref (60-80y)								
20-30y								
84% RH - SC samples of healthy & diseased volunteers								
AD (60-80y)								
Ref (60-80y)								
20-30y								
97% RH - SC samples of healthy & diseased volunteers								
AD (60-80y)								
Ref (60-80y)								
20-30y								

All samples were studied at different humidity conditions – dry (close to 0% RH), 84% RH and at 97% RH. The color schemes in the Table indicate changes in molecular dynamics of different carbon molecular segments as deduced from the relation between INEPT and CP intensities– INEPT > 4*CP; INEPT > 3*CP; INEPT > 2*CP; INEPT > CP; INEPT ≤ CP; no INEPT. For labeling and chemical shift of carbon segments, see Figure 1b and Table S1.

consistent with amino acids Tyr and His. In order to confirm the latter assignment of amino acids and rule out that these peaks originate from lipid components, we performed additional studies with samples composed of delipidized AD SC. Fig. 3 shows solid-state NMR spectra of AD delipidized SC in dry condition (Fig. 3 *a* and *b*) and at 97% RH (Fig. 3 *c* and *d*) for the low (left column) and high (right column) chemical shift regimes. For the samples prepared at 97% RH (Fig. 3 *c* and *d*), we again observe higher mobility for molecular segments corresponding to Gly, Ser, Tyr, and His in the AD delipidized SC samples compared with the reference SC sample (Fig. 2 *f* and *h*). For the dry state of AD delipidized SC samples (Fig. 3 *a* and *b*), no mobility was observed for any molecular segments, as inferred from the absence of INEPT peaks. It is important to note that the protocol employed to delipidize the SC does not remove lipids that are bound to the cornified envelope of the corneocytes. The presence of broad CP signals, combined with the absence of INEPT signals in the lipid region (e.g., 10–45 ppm), implies that the bound lipids exhibit

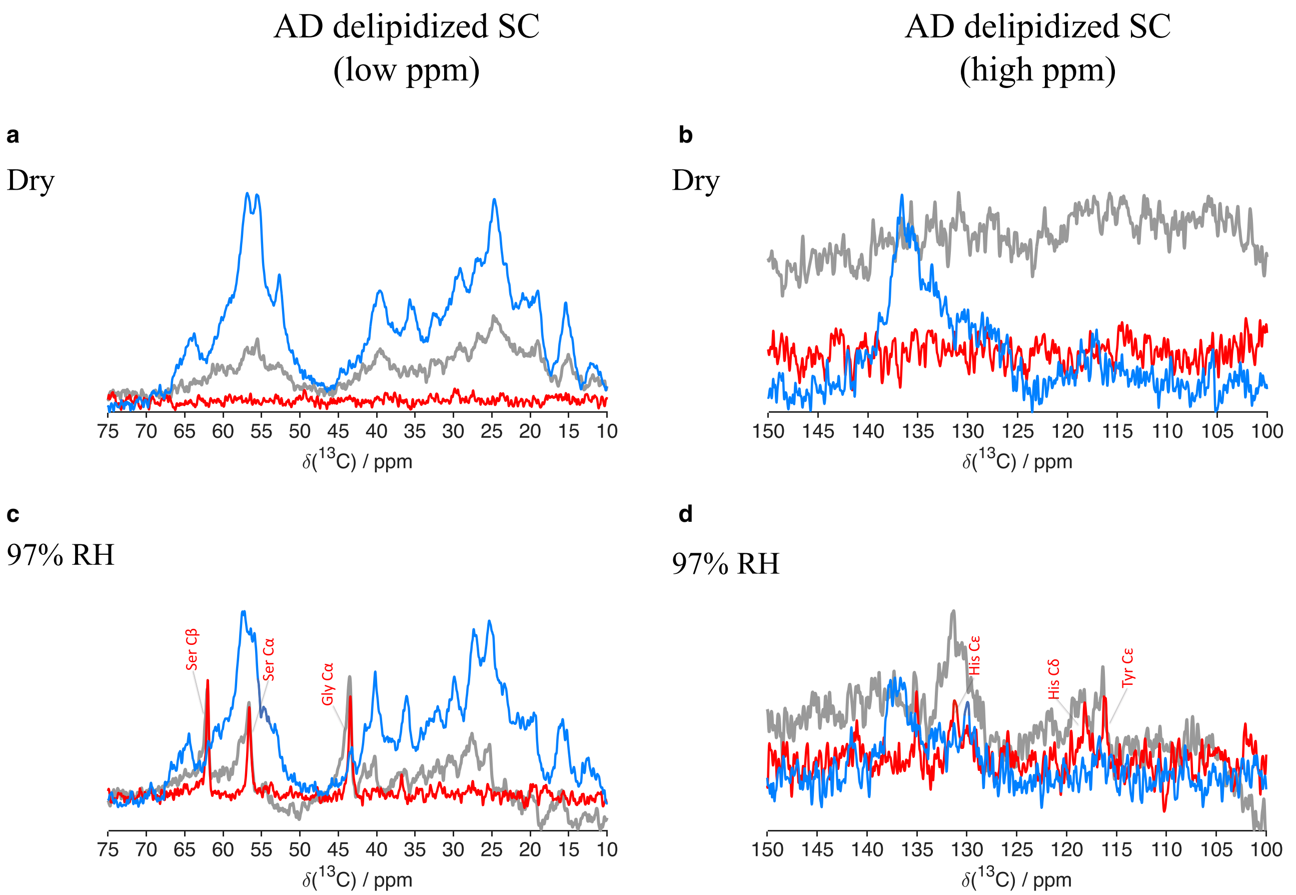

FIGURE 3 ^{13}C solid-state NMR spectra of AD delipidized SC samples. (*a* and *b*) Samples in dry (close to 0% RH) and (*c* and *d*) close to fully hydrated (97% RH) conditions at different chemical shift regimes. Left column: 10–75 ppm. Right column: 100–150 ppm. The individual DP (*gray*), CP (*blue*), and INEPT (*red*) spectrum are overlaid for comparison purpose. The resonance lines originating from various protein molecular segments are labeled in the spectra according to the assignment provided in Fig. 1 and Table S1.

rigid character with slow dynamics under the present experimental conditions.

Although NMR provides information on how the mobility of SC lipids and protein varies between the different samples and hydration conditions, WAXD experiments provide complementary structural information for the same samples. In particular, this technique is sensitive to detect changes in the more ordered structures, for example, the packing of the solid SC lipids (34). No detectable differences between the AD and reference SC could be resolved from the WAXD spectra at any hydration condition investigated (Fig. S4 *a* and *b*). From this, it is inferred that the structure of the solid lipid and protein assemblies is not affected by hydration or by the SC coming from AD or healthy individuals in any detectable way even though the proportions between the solid and mobile constituents differ.

Molecular mobility in SC from healthy individuals of different age

For all comparisons done above, we have compared the AD SC with SC samples from healthy individuals from the same age group (60–80 years old). As a last step of this study, we compared healthy SC from two different age groups, either 20–30 years old or 60–80 years old. The data are summarized in Fig. 4 and Table 2.

The comparison of SC molecular mobility in Fig. 4 revealed clear differences between the different age groups for all conditions investigated. First, we note that several previous studies have shown that the total lipid content decreases with age (71,72). This is consistent with the present observations of lower relative intensity of all peaks originating from lipid segments in all the NMR experiments. For dry conditions, there is no detectable INEPT signal from the samples from young individuals at any chemical shift, indicating that all SC lipid and protein components are rigid at this condition (Fig. 4 *a*). For intermediate and close to fully hydrated condition, on the other hand, we detected higher INEPT signal from several lipid segments for the SC from younger individuals compared with SC from the older age group (compare Fig. 4 *c* and *d*). For the protein components, we cannot distinguish any significant differences in the amino acid INEPT resonances when comparing young and elderly SC (Fig. 4 *c* and *d*). However, differences in the

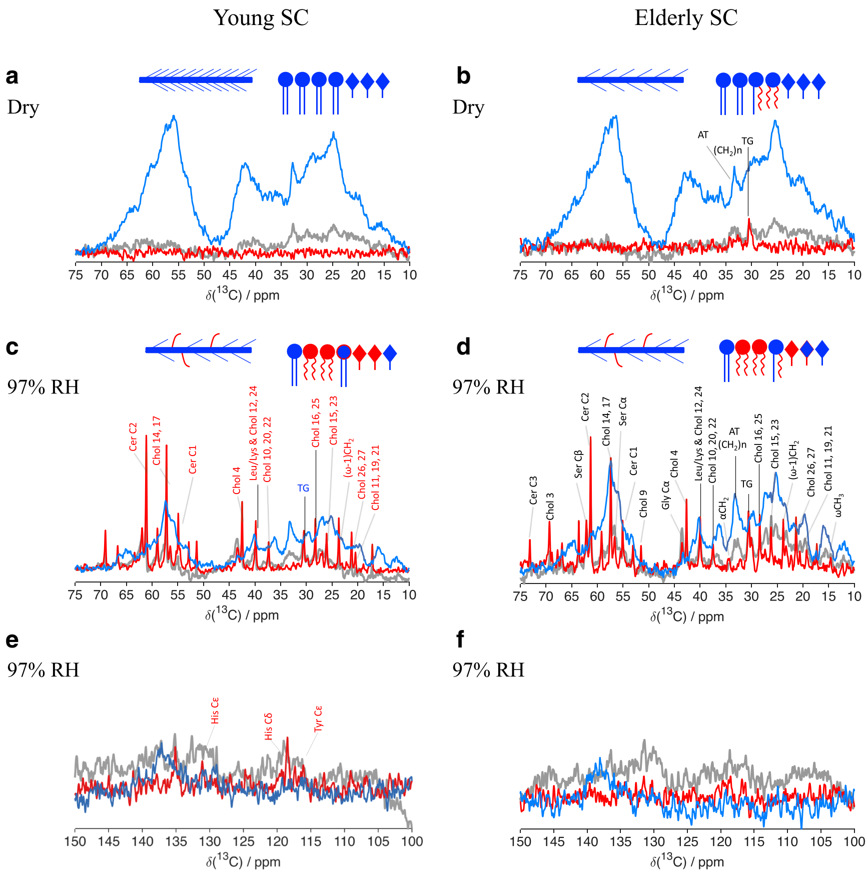

FIGURE 4 ^{13}C solid-state NMR study on SC from elderly and young individuals. The experiments were performed at three different hydration conditions: dry (close to 0% RH), intermediate (84% RH), and high (97% RH) humidity. The spectra for dry and high humidity conditions are shown here, whereas Fig. S2 presents the spectra for intermediate humidity (84% RH). The individual DP (*gray*), CP (*blue*), and INEPT (*red*) spectra are overlaid for comparison purpose. The resonance lines originating from various lipid and protein molecular segments are labeled according to the assignments provided in Fig. 1 and Table S1. In the comparisons, we again use the SC from the older age group as the reference sample. The red labeling in the spectra from the younger age group (*a*, *c*, and *e*) indicates an increase in INEPT/DP signal ratio as compared with the reference sample from the older age group at the same RH (*b*, *d*, and *f*). Analogously, blue labeling indicates reduced INEPT/DP signal ratio compared with the reference. The red labeling in the high ppm (100–150 ppm) region of the young SC spectrum at 97% RH (*e*) denotes amino acid signals from the keratin proteins that weren't seen in the reference SC spectrum (*f*) from the SC from elderly individuals. To facilitate interpretation, a cartoon representation showing molecular mobility and rigidity to the lipids and protein molecular components as observed in the solid-state NMR spectra is shown on top of the spectra. In the cartoons, blue and red indicate rigid and mobile molecular segments as evident from the CP (*blue*) and INEPT (*red*) spectra, respectively. The Cer and Chol headgroups in the cartoon are represented as round and diamond shapes, whereas straight and wiggly lines indicate rigid and mobile chain segments, respectively.

protein signals were detected when examining at chemical shifts ranging between 100 and 150 ppm (Fig. 4 *e* and *f*), where we only obtained discernable INEPT signals from His and Tyr amino acid segments in SC from the younger age group.

DISCUSSION

Herein, we demonstrate the potential of using solid-state NMR as a tool for characterization of complex biological material of human SC and distinguish between healthy and diseased skin through observations of molecular mobility. We characterize the molecular dynamics in SC harvested from AD patients and healthy individuals of various age groups. We observed distinct differences in the molecular dynamics of proteins and lipids in the SC of AD and healthy individuals. These variations, particularly in the balance between mobile and rigid molecular segments of SC lipids and protein keratin filaments at different hydration conditions and for SC from different origin, are intriguing and may provide further insights about lesional skin. Several studies have shown that the onset of AD is strongly associated with filaggrin deficiency (73–75). Filaggrin loss-of-function mutations associated with AD show significantly higher prevalence on pediatric conditions than young and elderly individuals (76), and their lipid profiles also differ markedly (77,78). Therefore, the observations made here on SC samples from elderly AD patients may not reflect the same scenario in pediatric AD. It has also been shown that acral plantar heel area is infrequently affected by AD compared with nonacral body sites, which are the typical sites of lesions (79). Additionally, lipid composition and corneodesmosome content differ between these body sites (79–81), which may also influence the quantitative outcomes.

In the present study, by using solid-state NMR, we have shown that the balance between fluid and solid lipid structures is altered by changes in hydration for both AD and healthy SC (Fig. 2). The solid-to-fluid melting transition naturally depends on the lipid composition. As a general rule, the fluid lipid phases are more likely to form in systems that contain lipids with short and/or unsaturated acyl-chains (82). If a certain lipid molecule is part of a fluid environment, the molecular dynamics of the whole molecule will most likely be high, including both the Cer headgroups

and the hydrocarbon chains. The Cer headgroup molecular dynamics will also depend on the overall dynamics in the interfacial region of the lipid lamellae, which depend on both the state of the chains and the surroundings, for example, the amount of freely diffusing water (36). The Chol component may distribute unevenly between solid and fluid lipid regions of the lipid lamellae (83) where its partitioning depends on the lipid acyl chain length and saturation (84). If there is a high amount of Chol dissolved in the fluid lipid regions, the overall molecular dynamics is expected to decrease due to Chol-induced ordering of the lipid acyl-chains, as previously characterized in detail for model lipid mixtures (21,85).

There are several studies showing AD-associated alterations in SC lipid composition that may be related to the here observed differences in SC lipid fluidity between the different SC samples. The levels of the enzymes involved in Cer and fatty acid biosynthesis are altered in AD skin as compared with healthy skin, which have consequences on the fatty acid chain composition and the balance between different Cer classes (75,86,87). There are also reports suggesting that the AD SC contains higher amounts of unsaturated FFAs and reduced amounts of the longer fatty acid chains compared with healthy SC (28,88). In addition, the levels of the EOS Cer are strongly reduced in AD SC (25,26,89). The Cer EOS is a ceramide esterified omega-hydroxy sphingosine linoleate with a very long chain, which is considered crucial for the formation of the long-periodicity lamellar structure, that may contain an embedded isotropic fluid layer containing the part of the chain with a double bond (18,29,90). The higher fraction of short chain lipids in the AD SC is consistent with the present observation of a significant fraction of mobile lipid chains even in the dry AD SC sample. The increased INEPT signal from Cer headgroups in both AD and reference SC at the highest RH infers that hydration induces melting of additional Cer lipids. The changes in the SC lipid solid/fluid balance as well as changes in the lipid lamellar arrangements likely impact the macroscopic properties of the SC. The observations of increased fluidity in AD SC lipids in dry and ambient conditions compared with the healthy SC lipids may explain previous observations of compromised SC barrier function and increased trans-epidermal water loss for AD skin compared with healthy skin (9,31,36,91,92).

When it comes to the protein components, we observe an overall higher mobility for several amino acids in the AD SC compared with the reference SC (Figs. 2 and 4). These effects can be due to changes in any protein, peptide, or amino acid components, which can be either the keratin filaments, NMF mixture, or any other protein. There are several studies showing that AD is associated with filaggrin deficiency (73–75). Filaggrin loss-of-function mutations associated with AD show significantly higher prevalence in pediatric conditions than in young and elderly individuals (76). Filaggrin is a protein that binds to and condenses the keratin cytoskeleton, causing the aggregation of keratin intermediate filaments into macrofibrils and thereby contributing to cellular compaction (93,94). This may explain the increased mobility in Ser and Gly, which are enriched in the SC protruding segments of the largely abundant keratin filaments in the corneocytes (35). However, we cannot rule out the possibility that these amino acids may also originate from filaggrin and other proteins in the cornified envelop or from free amino acids within the NMF. The breakdown products of filaggrin are also essential components of the NMF mixtures, which serve an important role in the SC by retaining fluidity in dehydrated condition (45,95). Reduced levels of NMF have previously been shown to cause reduced mobility in both SC lipid and protein components at lower relative humidities (46), which is opposite to the observed trends for both lipids and amino acids in AD SC. The observed differences can therefore not be explained only based on differences in NMF content in AD SC but are likely also related to changes in filament maturation and lipid composition.

Finally, we compare SC samples from different age groups. The SC from younger individuals shows less fluidity under dry conditions (Fig. 4). However, at intermediate and higher hydration levels, young SC exhibited a higher fluidity in both lipids and keratin proteins than elderly SC. There are previous observations that SC from younger individuals can hold more water compared with SC from older people (72,96,97), and that the skin from younger people is also less stiff with lower fracture stress (98). These macroscopic material properties of the SC may be related to differences in SC composition, structure, and molecular dynamics (24,34). Indeed, there are previous reports proposing that SC from younger individuals contains higher amounts of NMF (99) as well as CER EOS linoleate lipids compared with aged SC (72), both of which are associated with increased SC molecular mobility (29,46).

In summary, both lipids and proteins in the SC are predominantly in a rigid state at all hydration conditions. This is true for both AD SC and SC from healthy individuals from different age groups. Still, both healthy and AD SC samples contain a fraction of mobile lipids and protein, which is considered crucial to the macroscopic material properties of the upper layer of the skin (34). The lipids appear less mobile in AD samples in dry and ambient conditions. The mobile fraction in the SC lipids increases with increasing hydration, with a stronger response for AD SC compared with the reference SC. At the higher hydration levels, we also detect mobile fraction in the protein keratin filaments, with the strongest effects seen for the AD SC. These molecular differences between AD and healthy SC may lead to anomalies in macroscopic properties of the SC in terms of skin barrier functionality, trans-epidermal water loss, swelling in water, as well as mechanical properties. The detailed molecular information obtained from the current NMR studies can thus provide novel insights into

the material properties of healthy and diseased SC and may support the evaluation of therapeutic treatments with respect to the SC's molecular properties. This can have impact also in studies of other skin disorders.

DATA AND CODE AVAILABILITY

NMR and WAXD data have been deposited at Zenodo under the accession number https://doi.org/10.5281/zenodo.17794770 and are publicly available as of the article publication date.

ACKNOWLEDGMENTS

We are grateful to Cecilia Svedman and Ola Bergendorff for their assistance with the ethical application and sample collection procedure. We are also thankful to Göran Carlström for his assistance during NMR experiments. This work was supported by the Bo Rydin Foundation (grant no. F53/21 (E.S.)) and the Swedish Research Council (grant no. 2019-05296 (E.S.)) through regular grants. The Knut and Alie Wallenberg foundation funded the acquisition of the WAXD equipment.

AUTHOR CONTRIBUTIONS

Conceptualization: E.S., A.S., E.M., and Q.D.P.; formal analysis: E.M., E.S., A.S., Q.D.P., and D.T.; funding acquisition: E.S. and D.T.; writing – original draft preparation: E.M., E.S., A.S., and Q.D.P.; writing – review and editing: E.M., E.S., A.S., Q.D.P., and D.T.

DECLARATION OF INTERESTS

The authors state no conflict of interest.

SUPPORTING MATERIAL

Supporting material can be found online at https://doi.org/10.1016/j.bpj.2025.12.014.

REFERENCES

1. Langan, S. M., A. D. Irvine, and S. Weidinger. 2020. Atopic dermatitis. *Lancet.* 396:345–360.
2. Boguniewicz, M., and D. Y. M. Leung. 2011. Atopic dermatitis: a disease of altered skin barrier and immune dysregulation. *Immunol. Rev.* 242:233–246.
3. Stefanovic, N., and A. D. Irvine. 2024. Filaggrin and beyond: New insights into the skin barrier in atopic dermatitis and allergic diseases, from genetics to therapeutic perspectives. *Ann. Allergy Asthma Immunol.* 132:187–195.
4. Tsakok, T., R. Woolf, and C. Flohr. 2019. Atopic dermatitis: the skin barrier and beyond. *Br. J. Dermatol.* 180:464–474.
5. Agrawal, R., and J. A. Woodfolk. 2014. Skin barrier defects in atopic dermatitis. *Curr. Allergy Asthma Rep.* 14:433.
6. de Boer, F. L., H. F. van der Molen, and S. Kezic. 2023. Epidermal biomarkers of the skin barrier in atopic and contact dermatitis. *Contact Dermat.* 89:221–229.
7. Maeno, K. 2019. Direct Quantification of Natural Moisturizing Factors in Stratum Corneum using Direct Analysis in Real Time Mass Spectrometry with Inkjet-Printing Technique. *Sci. Rep.* 9:17789.
8. Jinnestål, C. L., E. Belfrage, and A. Sonesson. 2014. Skin barrier impairment correlates with cutaneous Staphylococcus aureus colonization and sensitization to skin-associated microbial antigens in adult patients with atopic dermatitis. *Int. J. Dermatol.* 53:27–33.
9. Yoshida, T., L. A. Beck, and A. De Benedetto. 2022. Skin barrier defects in atopic dermatitis: From old idea to new opportunity. *Allergol. Int.* 71:3–13.
10. Yang, G., J. K. Seok, and J. Y. Lee. 2020. Skin Barrier Abnormalities and Immune Dysfunction in Atopic Dermatitis. *Int. J. Mol. Sci.* 21:2867.
11. Menon, G. K., G. W. Cleary, and M. E. Lane. 2012. The structure and function of the stratum corneum. *Int. J. Pharm.* 435:3–9.
12. Elias, P. M., and D. S. Friend. 1975. The permeability barrier in mammalian epidermis. *J. Cell Biol.* 65:180–191.
13. Michaels, A. S., S. K. Chandrasekaran, and J. E. Shaw. 1975. Drug permeation through human skin: Theory and invitro experimental measurement. *AIChE J.* 21:985–996.
14. Starr, N. J., M. H. Khan, and D. J. Scurr. 2022. Elucidating the molecular landscape of the stratum corneum. *Proc. Natl. Acad. Sci. USA.* 119:e2114380119.
15. Norlén, L., I. Nicander, and B. Forslind. 1998. A new HPLC-based method for the quantitative analysis of inner stratum corneum lipids with special reference to the free fatty acid fraction. *Arch. Dermatol. Res.* 290:508–516.
16. Bouwstra, J. A., G. S. Gooris, and W. Bras. 1991. Structural Investigations of Human Stratum Corneum by Small-Angle X-Ray Scattering. *J. Invest. Dermatol.* 97:1005–1012.
17. Bouwstra, J. A., G. S. Gooris, and W. Bras. 1992. Structure of human stratum corneum as a function of temperature and hydration: A wide-angle X-ray diffraction study. *Int. J. Pharm.* 84:205–216.
18. Bouwstra, J. A., G. S. Gooris, and M. Ponec. 1998. Role of ceramide 1 in the molecular organization of the stratum corneum lipids. *J. Lipid Res.* 39:186–196.
19. Fandrei, F., T. Havrišák, and D. Huster. 2023. The intriguing molecular dynamics of Cer[EOS] in rigid skin barrier lipid layers requires improvement of the model. *J. Lipid Res.* 64:100356.
20. Engberg, O., A. Kováčik, and K. Vávrová. 2020. The Sphingosine and Acyl Chains of Ceramide [NS] Show Very Different Structure and Dynamics That Challenge Our Understanding of the Skin Barrier. *Angew. Chem. Int. Ed. Engl.* 59:17383–17387.
21. Mojumdar, E. H., G. S. Gooris, and J. A. Bouwstra. 2015. Phase behavior of skin lipid mixtures: the effect of cholesterol on lipid organization. *Soft Matter.* 11:4326–4336.
22. Blaess, M., and H. P. Deigner. 2019. Derailed Ceramide Metabolism in Atopic Dermatitis (AD): A Causal Starting Point for a Personalized (Basic) Therapy. *Int. J. Mol. Sci.* 20:3967.
23. Mojumdar, E. H., Z. Kariman, and J. A. Bouwstra. 2014. The role of ceramide chain length distribution on the barrier properties of the skin lipid membranes. *Biochim. Biophys. Acta.* 1838:2473–2483.
24. Sparr, E., S. Björklund, and D. Topgaard. 2023. The stratum corneum barrier – From molecular scale to macroscopic properties. *Curr. Opin. Colloid Interface Sci.* 67:101725.
25. Di Nardo, A., P. Wertz, and S. Seidenari. 1998. Ceramide and cholesterol composition of the skin of patients with atopic dermatitis. *Acta Derm. Venereol.* 78:27–30.
26. Imokawa, G., A. Abe, and A. Hidano. 1991. Decreased Level of Ceramides in Stratum Corneum of Atopic Dermatitis: An Etiologic Factor in Atopic Dry Skin? *J. Invest. Dermatol.* 96:523–526.
27. Fujii, M. 2021. The Pathogenic and Therapeutic Implications of Ceramide Abnormalities in Atopic Dermatitis. *Cells.* 10:2386.
28. van Smeden, J., M. Janssens, and J. A. Bouwstra. 2014. The importance of free fatty acid chain length for the skin barrier function in atopic eczema patients. *Exp. Dermatol.* 23:45–52.
29. Pham, Q. D., E. H. Mojumdar, and D. Topgaard. 2018. Solid and fluid segments within the same molecule of stratum corneum ceramide lipid. *Q. Rev. Biophys.* 51:e7.

30. Cork, M. J., S. G. Danby, and S. J. Ward. 2009. Epidermal Barrier Dysfunction in Atopic Dermatitis. *J. Invest. Dermatol.* 129:1892–1908.

31. Pilgram, G. S., D. C. Vissers, and H. K. Koerten. 2001. Aberrant Lipid Organization in Stratum Corneum of Patients with Atopic Dermatitis and Lamellar Ichthyosis. *J. Invest. Dermatol.* 117:710–717.

32. Uchino, T., D. Kamiya, and Y. Kagawa. 2023. Comparative analysis of intercellular lipid organization and composition between psoriatic and healthy stratum corneum. *Chem. Phys. Lipids.* 254:105305.

33. van Smeden, J., M. Janssens, and J. A. Bouwstra. 2014. Intercellular skin barrier lipid composition and organization in Netherton syndrome patients. *J. Invest. Dermatol.* 134:1238–1245.

34. Mojumdar, E. H., Q. D. Pham, and E. Sparr. 2017. Skin hydration: interplay between molecular dynamics, structure and water uptake in the stratum corneum. *Sci. Rep.* 7:15712.

35. Bjorklund, S., A. Nowacka, and D. Topgaard. 2013. Characterization of stratum corneum molecular dynamics by natural-abundance (1)(3)C solid-state NMR. *PLoS One.* 8:e61889.

36. Pham, Q. D., S. Björklund, and E. Sparr. 2016. Chemical penetration enhancers in stratum corneum - Relation between molecular effects and barrier function. *J. Control. Release.* 232:175–187.

37. Pham, Q. D., S. Gregoire, and E. Sparr. 2021. Skin hydration as a tool to control the distribution and molecular effects of intermediate polarity compounds in intact stratum corneum. *J. Colloid Interface Sci.* 603:874–885.

38. Pham, Q. D., D. Topgaard, and E. Sparr. 2017. Tracking solvents in the skin through atomically resolved measurements of molecular mobility in intact stratum corneum. *Proc. Natl. Acad. Sci. USA.* 114:E112–E121.

39. Björklund, S., J. Engblom, and E. Sparr. 2010. A water gradient can be used to regulate drug transport across skin. *J. Contr. Release.* 143:191–200.

40. Zhai, H., and H. I. Maibach. 2001. Effects of skin occlusion on percutaneous absorption: an overview. *Skin Pharmacol. Appl. Skin Physiol.* 14:1–10.

41. Wu, K. S., W. W. van Osdol, and R. H. Dauskardt. 2006. Mechanical properties of human stratum corneum: Effects of temperature, hydration, and chemical treatment. *Biomaterials.* 27:785–795.

42. Vyumvuhore, R., A. Tfayli, and A. Baillet-Guffroy. 2015. The relationship between water loss, mechanical stress, and molecular structure of human stratum corneum ex vivo. *J. Biophotonics.* 8:217–225.

43. Berkey, C., K. Biniek, and R. H. Dauskardt. 2021. Predicting hydration and moisturizer ingredient effects on mechanical behavior of human stratum corneum. *Extreme Mech. Lett.* 46:101327.

44. Guo, S., Y. Domanov, and G. S. Luengo. 2019. Anisotropic cellular forces support mechanical integrity of the Stratum Corneum barrier. *J. Mech. Behav. Biomed. Mater.* 92:11–23.

45. Björklund, S., J. M. Andersson, and E. Sparr. 2014. Stratum corneum molecular mobility in the presence of natural moisturizers. *Soft Matter.* 10:4535–4546.

46. Gunnarsson, M., E. H. Mojumdar, and E. Sparr. 2021. Extraction of natural moisturizing factor from the stratum corneum and its implication on skin molecular mobility. *J. Colloid Interface Sci.* 604:480–491.

47. Matlahov, I., and P. C. A. van der Wel. 2018. Hidden motions and motion-induced invisibility: Dynamics-based spectral editing in solid-state NMR. *Methods.* 148:123–135.

48. Topgaard, D. 2023. Skin, soap, and spaghetti: investigations of co-existing solid and liquid phases in organic materials using solid-state NMR with dynamics-based spectral editing. *Appl. Chem.* 95:1075–1089.

49. Nowacka, A., N. A. Bongartz, and D. Topgaard. 2013. Signal intensities in 1H–13C CP and INEPT MAS NMR of liquid crystals. *J. Magn. Reson.* 230:165–175.

50. Nowacka, A., P. C. Mohr, and D. Topgaard. 2010. Polarization transfer solid-state NMR for studying surfactant phase behavior. *Langmuir.* 26:16848–16856.

51. Mojumdar, E. H., and E. Sparr. 2021. The effect of pH and salt on the molecular structure and dynamics of the skin. *Colloids Surf. B Biointerfaces.* 198:111476.

52. Pham, Q. D., G. Carlström, and D. Topgaard. 2020. Quantification of the amount of mobile components in intact stratum corneum with natural-abundance 13C solid-state NMR. *Phys. Chem. Chem. Phys.* 22:6572–6583.

53. Yagi, N., K. Aoyama, and N. Ohta. 2020. Microbeam X-ray diffraction study of lipid structure in stratum corneum of human skin. *PLoS One.* 15:e0233131.

54. Bjorklund, S., T. Ruzgas, and J. Engblom. 2013. Skin membrane electrical impedance properties under the influence of a varying water gradient. *Biophys. J.* 104:2639–2650.

55. Björklund, S., A. Nowacka, and D. Topgaard. 2013. Characterization of Stratum Corneum Molecular Dynamics by Natural-Abundance 13C Solid-State NMR. *PLoS One.* 8:e61889.

56. Wertz, P. W., and D. T. Downing. 1987. Covalently bound omega-hydroxyacylsphingosine in the stratum corneum. *Biochim. Biophys. Acta.* 917:108–111.

57. Swartzendruber, D. C., P. W. Wertz, and D. T. Downing. 1987. Evidence that the corneocyte has a chemically bound lipid envelope. *J. Invest. Dermatol.* 88:709–713.

58. Pines, A., M. G. Gibby, and J. S. Waugh. 1972. Proton-Enhanced Nuclear Induction Spectroscopy. A Method for High Resolution NMR of Dilute Spins in Solids. *J. Chem. Phys.* 56:1776–1777.

59. Morris, G. A., and R. Freeman. 1979. Enhancement of nuclear magnetic resonance signals by polarization transfer. *J. Am. Chem. Soc.* 101:760–762.

60. Bennett, A. E., C. M. Rienstra, and R. G. Griffin. 1995. Heteronuclear decoupling in rotating solids. *J. Chem. Phys.* 103:6951–6958.

61. Metz, G., X. L. Wu, and S. O. Smith. 1994. Ramped-Amplitude Cross Polarization in Magic-Angle-Spinning NMR. *J. Magn. Reson., Ser. A.* 110:219–227.

62. Burum, D. P., and R. R. Ernst. 1980. Net polarization transfer via a J-ordered state for signal enhancement of low-sensitivity nuclei. *J. Magn. Reson.* 39:163–168.

63. van Beek, J. D. 2007. matNMR: A flexible toolbox for processing, analyzing and visualizing magnetic resonance data in Matlab®. *J. Magn. Reson.* 187:19–26.

64. Bouwstra, J. A., G. S. Gooris, and M. Ponec. 1996. Phase behavior of isolated skin lipids. *J. Lipid Res.* 37:999–1011.

65. Alonso, A., N. C. Meirelles, and M. Tabak. 1996. Water increases the fluidity of intercellular membranes of stratum corneum: correlation with water permeability, elastic, and electrical resistance properties. *J. Invest. Dermatol.* 106:1058–1063.

66. Wildnauer, R. H., J. W. Bothwell, and A. B. Douglass. 1971. Stratum corneum biomechanical properties I. influence of relative humidity on normal and extracted human stratum corneum. *J. Invest. Dermatol.* 56:72–78.

67. Papir, Y. S., K. H. Hsu, and R. H. Wildnauer. 1975. The mechanical properties of stratum corneum. *Biochim. Biophys. Acta.* 399:170–180.

68. Warner, R. R., K. J. Stone, and Y. L. Boissy. 2003. Hydration disrupts human stratum corneum ultrastructure. *J. Invest. Dermatol.* 120:275–284.

69. Bouwstra, J. A., A. de Graaff, and A. C. van Aelst. 2003. Water distribution and related morphology in human stratum corneum at different hydration levels. *J. Invest. Dermatol.* 120:750–758.

70. Caspers, P. J., G. W. Lucassen, and G. J. Puppels. 2001. In vivo confocal Raman microspectroscopy of the skin: noninvasive determination of molecular concentration profiles. *J. Invest. Dermatol.* 116:434–442.

71. Starr, N. J., D. J. Johnson, and D. J. Scurr. 2016. Age-Related Changes to Human Stratum Corneum Lipids Detected Using Time-of-Flight Secondary Ion Mass Spectrometry Following in Vivo Sampling. *Anal. Chem.* 88:4400–4408.

72. Rogers, J., C. Harding, and A. Rawlings. 1996. Stratum corneum lipids: the effect of ageing and the seasons. *Arch. Dermatol. Res.* 288:765–770.

73. Cabanillas, B., and N. Novak. 2016. Atopic dermatitis and filaggrin. *Curr. Opin. Immunol.* 42:1–8.

74. Palmer, C. N. A., A. D. Irvine, and W. H. I. McLean. 2006. Common loss-of-function variants of the epidermal barrier protein filaggrin are a major predisposing factor for atopic dermatitis. *Nat. Genet.* 38:441–446.

75. Boer, D. E. C., J. van Smeden, and J. A. Bouwstra. 2020. Skin of atopic dermatitis patients shows disturbed β-glucocerebrosidase and acid sphingomyelinase activity that relates to changes in stratum corneum lipid composition. *Biochim. Biophys. Acta. Mol. Cell Biol. Lipids.* 1865:158673.

76. Virolainen, S. J., L. Satish, and L. C. Kottyan. 2024. Filaggrin loss-of-function variants are associated with atopic dermatitis phenotypes in a diverse, early-life prospective cohort. *JCI Insight.* 9:e178258.

77. Zhang, Y., H. Gu, and W. Yan. 2023. Trajectory of stratum corneum lipid subclasses in the first year of life and associations with atopic dermatitis: A prospective cohort study. *Ped All Immun.* 34:e14045.

78. Facheris, P., J. C. Da Rosa, and E. Guttman-Yassky. 2023. Age of onset defines two distinct profiles of atopic dermatitis in adults. *Allergy.* 78:2202–2214.

79. Merleev, A. A., S. T. Le, and E. Maverakis. 2022. Biogeographic and disease-specific alterations in epidermal lipid composition and single-cell analysis of acral keratinocytes. *JCI Insight.* 7:e159762.

80. Rawlings, A. V., M. E. Lane, and R. Voegeli. 2024. The importance of stratum corneum ω-linoleoyloxyacylceramides in human skin barrier health: their biochemistry, processing enzymes and metabolites involved in corneocyte lipid envelope maturation. *Int J Cosm Sci.* 46:623–642.

81. Egelrud, T., and A. Lundström. 1991. Intercellular lamellar lipids in plantar stratum corneum. *Acta Derm. Venereol.* 71:369–372.

82. Small, D. M. 1986. The Physical Chemistry of Lipids : From Alkanes to Phospholipids. Plenum Press, New York.

83. Mojumdar, E. H., D. Groen, and J. A. Bouwstra. 2013. Localization of cholesterol and fatty acid in a model lipid membrane: a neutron diffraction approach. *Biophys. J.* 105:911–918.

84. Ramos, A. P., and M. Lafleur. 2015. Chain Length of Free Fatty Acids Influences the Phase Behavior of Stratum Corneum Model Membranes. *Langmuir.* 31:11621–11629.

85. Ferreira, T. M., F. Coreta-Gomes, and D. Topgaard. 2013. Cholesterol and POPC segmental order parameters in lipid membranes: solid state 1H–13C NMR and MD simulation studies. *Phys. Chem. Chem. Phys.* 15:1976–1989.

86. Danso, M., W. Boiten, and J. Bouwstra. 2017. Altered expression of epidermal lipid bio-synthesis enzymes in atopic dermatitis skin is accompanied by changes in stratum corneum lipid composition. *J. Dermatol. Sci.* 88:57–66.

87. Rabionet, M., K. Gorgas, and R. Sandhoff. 2014. Ceramide synthesis in the epidermis. *Biochim. Biophys. Acta.* 1841:422–434.

88. Chermprapai, S., F. Broere, and J. A. Bouwstra. 2018. Altered lipid properties of the stratum corneum in Canine Atopic Dermatitis. *Biochim. Biophys. Acta. Biomembr.* 1860:526–533.

89. Yamamoto, A., S. Serizawa, and Y. Sato. 1991. Stratum corneum lipid abnormalities in atopic dermatitis. *Arch. Dermatol. Res.* 283:219–223.

90. Paz Ramos, A., G. Gooris, and M. Lafleur. 2018. Evidence of hydrocarbon nanodrops in highly ordered stratum corneum model membranes. *J. Lipid Res.* 59:137–143.

91. Sahle, F. F., T. Gebre-Mariam, and R. H. H. Neubert. 2015. Skin Diseases Associated with the Depletion of Stratum Corneum Lipids and Stratum Corneum Lipid Substitution Therapy. *Skin Pharmacol. Physiol.* 28:42–55.

92. Janssens, M., J. van Smeden, and J. A. Bouwstra. 2012. Increase in short-chain ceramides correlates with an altered lipid organization and decreased barrier function in atopic eczema patients[S]. *J. Lipid Res.* 53:2755–2766.

93. Sandilands, A., C. Sutherland, and W. H. I. McLean. 2009. Filaggrin in the frontline: role in skin barrier function and disease. *J. Cell Sci.* 122:1285–1294.

94. Moosbrugger-Martinz, V., C. Leprince, and S. Dubrac. 2022. Revisiting the Roles of Filaggrin in Atopic Dermatitis. *Int. J. Mol. Sci.* 23:5318.

95. Scott, I. R., and C. R. Harding. 1986. Filaggrin breakdown to water binding compounds during development of the rat stratum corneum is controlled by the water activity of the environment. *Dev. Biol.* 115:84–92.

96. Rawlings, A. V. 2017. The Stratum Corneum and Aging. *In* Textbook of Aging Skin. M. A. Farage, K. W. Miller, and H. I. Maibach, eds Springer Berlin Heidelberg, Berlin, Heidelberg, pp. 67–90.

97. Boireau-Adamezyk, E., A. Baillet-Guffroy, and G. N. Stamatas. 2021. The stratum corneum water content and natural moisturization factor composition evolve with age and depend on body site. *Int. J. Dermatol.* 60:834–839.

98. Biniek, K., J. Kaczvinsky, and R. H. Dauskardt. 2015. Understanding age-induced alterations to the biomechanical barrier function of human stratum corneum. *J. Dermatol. Sci.* 80:94–101.

99. Choe, C., J. Schleusener, and M. E. Darvin. 2018. Age related depth profiles of human Stratum Corneum barrier-related molecular parameters by confocal Raman microscopy in vivo. *Mech. Ageing Dev.* 172:6–12.

Biophysical Journal, Volume *125*

Supplemental information

Molecular dynamics in the stratum corneum of plantar heels in atopic dermatitis patients

Enamul Mojumdar, Andreas Sonesson, Quoc Dat Pham, Daniel Topgaard, and Emma Sparr

SUPPLEMENTARY INFORMATION

Table S1. ^{13}C solid-state NMR peak assignments. Peak assignments with their corresponding chemical shifts are provided for all major SC lipid and protein molecular segments.

**SC lipids and protein**	**Regions**	**^{13}C segments**	**$\delta(^{13}C)$ / ppm**
Lipids (Ceramide, Cholesterol and Free fatty acids)	Chain terminal	ωCH_3	14.6
		$(\omega\text{-}1)CH_2$	23.3
		$(\omega\text{-}2)CH_2$	32.0 – 32.7
	Chain mid	$(CH_2)_n$ TG	30.5
	Chain interface	αCH_2	34.8
	Ceramide head-group	C1	55.4
		C2	61.3
		C4	135
	Chol	3	70.1
		4	42.7
		9	51.5
		14 & 17	57
		12 & 24	40.4
Protein (keratin filaments)	Terminal	Gly Cα	43.7
		Ser Cα	56.6
		Ser Cβ	62.1
		Leu Cβ & Lys Cε	40.6
		Tyr Cε	116
		His Cδ	118
		His Cε	132

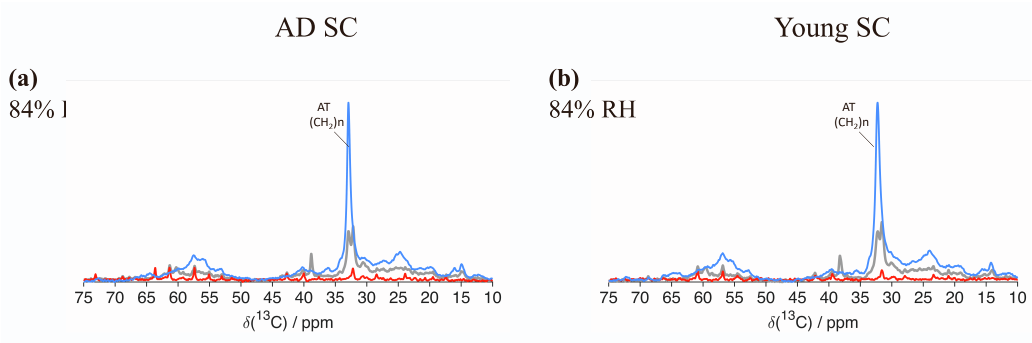

Figure S1. SC contaminated samples as observed with solid-state NMR. (a, b) SC contaminated sample from AD heel region (60-80y) and younger age group (20-30y) at 84% RH. In Figure S1a, large TG peak at approx. 30.5 ppm and other contributions in the INEPT is probably due to skin surface sebum lipids/phospholipids which are in fluid state at ambient condition. In Figure S1b, high AT peak centered around 32.5 ppm might be due to the presence of petrolatum/waxes in creams/ formulations applied onto the skin.

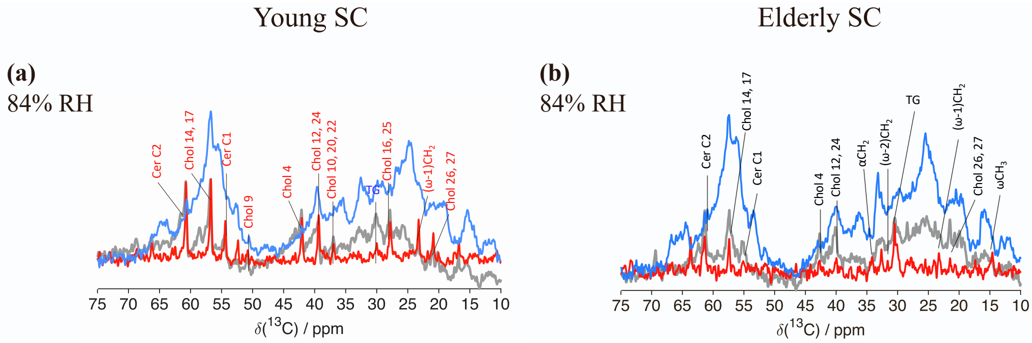

Figure S2. ^{13}C solid-state NMR study on young and elderly SC. (a, b) Samples from young and elderly SC at intermediate hydration regime corresponds to 84% RH. The individual DP (gray), CP (blue) and INEPT (red) spectrum is overlaid in all experiments for comparison purpose. The resonance lines originating from various lipid and protein molecular segments are labelled in the spectra according to the assignments provided in Figure 1 and table S1. The labelling in black in the elderly spectra (b) was used as a reference when comparing with young spectra (a). In the young spectra, the blue assignments indicate reduced signal ratio when comparing with reference for INEPT/DP signal intensities. The red labelling on the other hand indicate that a signal is increased when comparing with elderly spectra in the ratio of INEPT/DP.

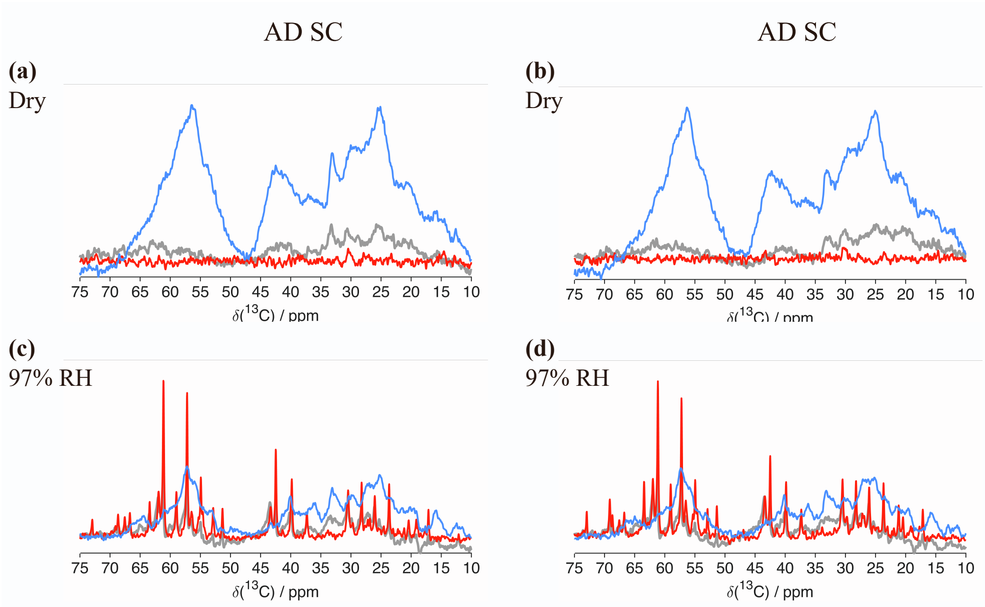

Figure S3. ^{13}C solid-state NMR study on AD SC (age 77 year). (a, c) AD sample in dry and high hydration regime corresponds to 97% RH. The individual DP (gray), CP (blue) and INEPT (red) spectrum is overlaid in all experiments for comparison purpose. The resonance lines originating from various lipid and protein molecular segments in dry and at 97% RH are very similar to the spectra shown in Figure 2 (a – dry & e – 97% RH) for AD SC and is also provided here for comparison purpose without assignments (b – dry & d – 97% RH).

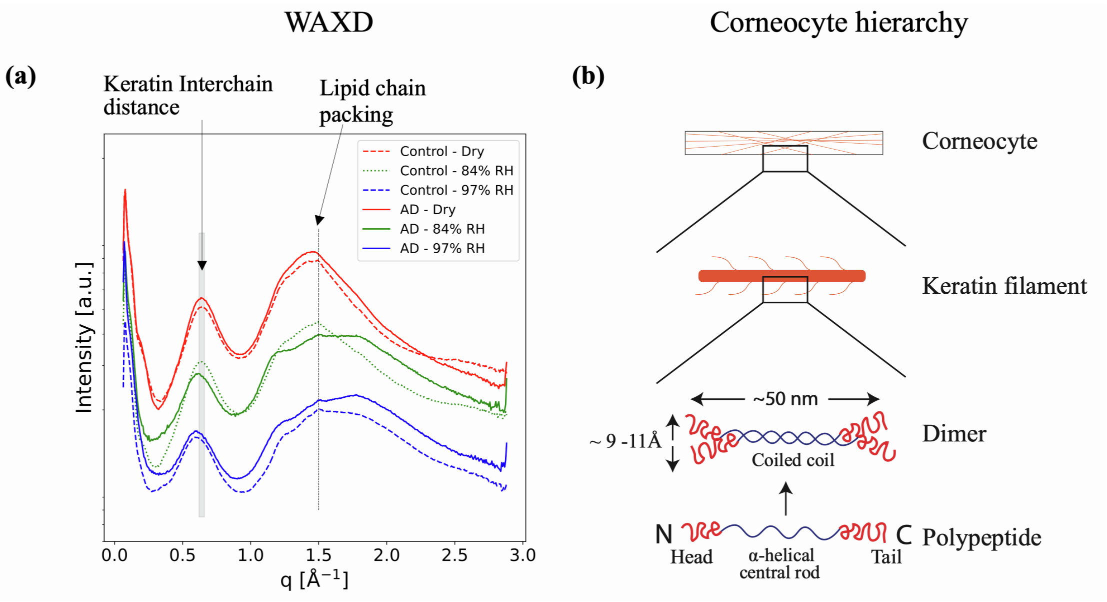

Figure S4. WAXD spectra on skin samples. (a) WAXD spectra of reference (dashed line) and AD (solid line) SC samples measured at 32 °C and at different ambient RH levels. The different colors in the spectra indicate the different hydration levels as controlled by the RH in the sample surrounding and indicated in the Figure legend. The peaks in the shaded region originate from the keratin interchain distance comprised of coiled coil dimer as depicted in (b) for corneocyte (delipidized SC) structural hierarchy. The vertical dashed line in the WAXD spectra indicate peaks arising from the lipid acyl chain packing and have not shifted due to the change in sample hydration levels.
